# Supplementary material for: Heat adaptation of phage T7 under an extended genetic code
Source: Virus Evol. 2021 Dec 1;7(2):veab100. doi: 10.1093/ve/veab100 (PMC8923235; doi:10.1093/ve/veab100)
Supplement: veab100_Supp [file veab100_supp.zip › Sequencing_Analysis.zip!/Sequencing_Analysis/A1/evidence/JC_54.html]

BRESEQ :: Evidence


| New junction evidence | | | | | | | | | | | |
| --- | --- | --- | --- | --- | --- | --- | --- | --- | --- | --- | --- |
|  | | seq id | position | reads (cov) | reads (cov) | score | skew | freq | annotation | gene | product |
| \* | ? | NC\_001604.1 | = 10002 | 2433 (0.790) | 15 (0.000) | 14/594 | 38.4 | 0.51% | intergenic (–/–) | *–/–* | –/– |
|  | ? | NC\_001604.1 | 10186 = | 3422 (1.120) |  |  |  |  | intergenic (–/–) | *–/–* | –/– |
| Rejected: Coverage evenness skew score above cutoff. | | | | | | | | | | | |
| Rejected: Frequency below/above cutoff threshold. | | | | | | | | | | | |

Only 100 of 117 total aligned reads displayed.  

`ACCTTTGGTGGCGGTGAAGACGATTGGGCTGACGAAGTTGAAGAGAACGGCTATGTTGCCTCTGGTTCTGCCAAAGCGAGCAAACCACGCGACGAAGAAAGCTGGGACGAAGACGACGAAGAGTCCGAGGAAGCAGACGAAGACGGAGACTTCTAAGTGGAACTGCGGGAGAAAATCCTTGAGCGAATCAAGGTGACTTCCTCTGGGTGTTGGGAGTGGCAGGGCGCTACGAACAATAAAGGGTACGGGCAGGTGTGGTGCAGCAATACCGGAAAGGTTGTCTACTGTCATCGCGTAATGTC‑‑‑‑‑‑‑‑‑‑‑‑‑‑‑‑‑‑‑‑‑‑‑‑‑‑‑‑‑‑‑‑‑‑‑‑‑‑‑‑‑‑‑‑‑‑‑‑‑‑‑‑‑‑‑‑‑‑‑‑‑‑‑‑‑‑‑‑‑‑‑‑‑‑‑‑‑‑‑‑‑‑‑‑‑‑‑‑‑‑‑‑‑‑‑‑‑‑‑‑‑‑‑‑‑‑‑‑‑‑‑‑‑‑‑‑‑‑‑‑‑‑‑‑‑‑‑‑‑‑‑‑‑‑‑‑‑‑‑‑‑‑‑‑‑‑‑‑‑‑‑‑‑‑‑‑‑‑‑‑‑‑‑‑‑‑‑‑‑‑‑‑‑‑‑‑‑‑‑‑‑‑‑‑‑‑‑‑‑‑‑‑‑‑‑‑‑‑‑‑‑‑‑‑‑‑‑‑‑‑‑‑‑‑‑‑‑‑‑‑‑‑‑‑‑‑‑‑‑‑‑‑‑‑‑‑‑‑‑‑‑‑‑‑‑‑‑‑‑‑‑‑‑‑‑‑‑‑‑‑‑‑‑‑‑‑‑‑‑‑‑‑‑‑‑‑‑‑‑‑‑‑‑‑‑‑‑‑‑‑‑‑‑‑‑‑‑‑‑‑  >  NC_001604.1/9701‑10002`  
`‑‑‑‑‑‑‑‑‑‑‑‑‑‑‑‑‑‑‑‑‑‑‑‑‑‑‑‑‑‑‑‑‑‑‑‑‑‑‑‑‑‑‑‑‑‑‑‑‑‑‑‑‑‑‑‑‑‑‑‑‑‑‑‑‑‑‑‑‑‑‑‑‑‑‑‑‑‑‑‑‑‑‑‑‑‑‑‑‑‑‑‑‑‑‑‑‑‑‑‑‑‑‑‑‑‑‑‑‑‑‑‑‑‑‑‑‑‑‑‑‑‑‑‑‑‑‑‑‑‑‑‑‑‑‑‑‑‑‑‑‑‑‑‑‑‑‑‑‑‑‑‑‑‑‑‑‑‑‑‑‑‑‑‑‑‑‑‑‑‑‑‑‑‑‑‑‑‑‑‑‑‑‑‑‑‑‑‑‑‑‑‑‑‑‑‑‑‑‑‑‑‑‑‑‑‑‑‑‑‑‑‑‑‑‑‑‑‑‑‑‑‑‑‑‑‑‑‑‑‑‑‑‑‑‑‑‑‑‑‑‑‑‑‑‑‑‑‑‑‑‑‑‑‑‑‑‑‑‑‑‑‑‑‑‑‑‑‑‑‑‑‑‑‑‑‑‑‑‑‑‑‑‑‑‑‑‑‑‑‑‑‑‑‑‑‑‑‑‑gtcCAATGTATCCTTAGCTCGCACCTATGGTGTCTCCCAACAGACTATTTGTGATATACGCAAAGGGAGGCGACATGGCAGGTTACGGCGCTAAAGGAATCCGAAAGGTTGGAGCGTTTCGCTCTGGCCTAGAGGACAAGGTTTCAAAGCAGTTGGAATCAAAAGGTATTAAATTCGAGTATGAAGAGTGGAAAGTGCCTTATGTAATTCCGGCGAGCAATCACACTTACACTCCAGACTTCTTACTTCCAAACGGTATATTCGTTGAGACAAAGGGTCTGTGGGAAAGCGATGATAGAAAGA  >  NC_001604.1/10186‑10485`  

                                                                                                                                                                                                                                                                                                                                                                                                                                                                                                                                                                                                                            
`gagatgtgtataagagccagCGATTGGGCTGACGAAGTTGAAGAGAACGGCTATGTTGCCTCTGGTTCTGCCAAAGCGGGCAAACCACGCGACGAAGAAAGCTGGGACGAAGACGACGAAGAGTCCGAGGAAGCAGACGAAGACGGAGACTTCTAAGTGGAACTGCGGGAGAAAATCCTTGAGCGAATCAAGGTGACTTCCTCTGGGTGTTGGGAGTGGCAGGGCGCTACGAACAATAAAGGGTACGGGCAGGTGTGGTGCAGCAATACCGGAAAGGTTGTCTACTGTCATCGCGTAATGT                                                                                                                                                                                                                                                                                                               <  1:357310/281‑1`  
`cCCTTTGGTGGCGGTGAAGACGATTGGGCTGACGAAGTTGAAGAGAACGGCGATGTTGCCTCTGGTTCTGCCAAAGCGAGCAAACCACGCGACGAAGAAAGCTGTGACGAAGACGACGAAGAGTCCGATGAAGCAGACGAAGACGTAGACTTCTAAGTGGAACTGCGGGAGAAAATCCTTGAGCGAATCAAGGTGACTTCCTCTGGGTGTTGTGAGTGGCAGGGCGCTACGAACAATAAAGGGTACGGGCAGGTGTGGTGCAGCAATACCGGAAAGGTTGTCTACTGTCATCGCGTAATGT                                                                                                                                                                                                                                                                                                               <  1:312970/300‑1`  
`atgggtataagggacagAGACGATTGGGATGACGAAGTTGAAGAGAACGGCTATGTTGCCTCTGGTTCTGCCAAAGCGAGCAAACCACGCGACGAAGAAAGCTGGGACGAAGACGACGAAGAGTCCGAGGAAGCAGACGAAGACGGAGACTTCTAAGTGGAACTGCGGGAGAAAATCCTTGAGCGAATCAAGGTGACTTCCTCTGGGTGTTGGGAGTGGCAGGGCGCTACGAACAATAAAGGGTACGGGCAGGTGTGGTGCAGCAATACCGGAAAGGTTGTCTACTGTCATCGCGTAATGT                                                                                                                                                                                                                                                                                                               <  1:283941/284‑1`  
`ACCTTTGGTTGCGGTGAAGACGATTGGGCTGACGAAGTTGAAGAGAACGGCTATGTTGCCTCTGGTTCTGCCAAAGCGAGCAAACCACGCGACGAAGAAAGCTGGGACGAAGACGACGAAGAGTCCGAGGAAGCAGACGAAGACGGAGACTTCTAAGTGGAACTGCGGGAGAAAATCCTTGCGCGAATCAAGGTGACTTCCTCTGGGTGTTGGGAGTGGCAGGGCGCTACGAACAATAAAGGGTACGGGCAGGTGTGGTGCAGCAATACCGGAAAGGTTGTCTACTGTCATCGCGTAATGT                                                                                                                                                                                                                                                                                                               <  1:441461/301‑1`  
`ACCTTTGGTGGCGGTGAAGACGATTGGGCTGACGAATTTGAAGAGAACGGCTATGTTGCCTCTGGTTCTGCCAAAGCGAGCAAACCACGCGACGAAGAAAGCTGGGACGAAGACGACGAAGAGTCCGAGGAAGCAGACGAAGACGGAGACTTCTACGTGGAACTGCGTGAGAAAATCCTTGAGCGCATCAACGTGACTTCCTCTTCGTGTTGGGTGTGGCAGGTCGCTACGAACAATAATGTCTACGGGCCGGTGTGATGCACCAATACCGACAAGGTTGTCTACTGTCATCGCGTAATGT                                                                                                                                                                                                                                                                                                               >  1:47158/1‑301`  
`ACCTTTGGTGGCGGTGAAGACGATTGGGCTGACGAAGTTGAAGAGAACTGCTATGTTGCCTCTGGTTCTGCCAAAGCGAGCAAACCACGCGACGAAGAAAGCTGGGACGAAGACGACGAAGAGTCCGAGGAAGCAGACGAAGACGGAGACTTCTAAGTGGAACTGCGGGAGAAAATCCTTGAGCGAATCAAGGTGACTTCCTCTGGGTGTTGGGAGTGGCAGGGTGCTACGAACAATAAAGGGTACGGGCAGGTGTGGTGCAGCAATACCGGAAAGGTTGTCTACTGTCATCGCGTAATGT                                                                                                                                                                                                                                                                                                               >  1:91454/1‑301`  
`ACCTTTGGTGGCGGTGAAGACGATTGGGCTGACGAAGTTGAAGAGAACGGCTATGTTGCCTCTGGTTCTGCCAAAGCGAGCAAACCACGCGACGAAGAAAGCTGGGACGAAGACGACGAAGAGTCCGAGGAAGCAGACGAAGACGGAGACTTCTAAGTGGAACTTCGGGAGAAAATCCTTGAGCGAATCAAGGTGACTTCCTCTGGGTGTTGGGAGTGGCAGGGCGCTACGAACAATAAAGGGTACGGGCAGGTGTGGTGCAGCAATACCGGAAAGGTTGTCTACTGTCATCGCGTAATGT                                                                                                                                                                                                                                                                                                               >  1:362972/1‑301`  
`ACCTTTGGTGGCGGTGAAGACGATTGGGCTGACGAAGTTGAAGAGAACGGCTATGTTGCCTCTGGTTCTGCCAAAGCGAGCAAACCACGCGACGAAGAAAGCTGGGACGAAGACGACGAAGAGTCCGAGGAAGCAGACGAAGACGGAGACTTCTAAGTGGAACTGCGGGAGAAAATCCTTGAGCGAATCAAGGTGACTTCCTCTGGGTGTTGGGAGTGGCAGGGCGCTACGAACAATAAAGGGTACGGGCAGGTGTGGTGCAGCAATACCGGAAAGGTTGTCTACTGTCATCGCGTAATGc                                                                                                                                                                                                                                                                                                               >  1:146826/1‑300`  
`ACCTTTGGTGGCGGTGAAGACGATTGGGCTGACGAAGTTGAAGAGAACGGCTATGTTGCCTCTGGTTCTGCCAAAGCGAGCAAACCACGCGACGAAGAAAGCTGGGACGAAGACGACGAAGAGTCCGAGGAAGCAGACGAAGACGGAGACTTCTAAGTGGAACTGCGGGAGAAAATCCTTGAGCGAATCAAGGTGACTTCCTCTGGGTGTTGGGAGTGGCAGGGCGCTACGAACAATAAAGGGTACGGGCAGGTGTGGTGCAGCAATACCGGAAAGGTTGTCTACTGTCATCGCGTAATGT                                                                                                                                                                                                                                                                                                               >  1:232056/1‑301`  
`ACCTTTGGTGGCGGTGAAGACGATTGGGCTGACGAAGTTGAAGAGAACGGCTATGTTGCCTCTGGTTCTGCCAAAGCGAGCAAACCACGCGACGAAGAAAGCTGGGACGAAGACGACGAAGAGTCCGAGGAAGCAGACGAAGACGGAGACTTCTAAGTGGAACTGCGGGAGAAAATCCTTGAGCGAATCAAGGTGACTTCCTCTGGGTGTTGGGAGTGGCAGGGCGCTACGAACAATAAAGGGTACGGGCAGGTGTGGTGCAGCAATACCGGAAAGGTTGTCTACTGTCATCGCGTAATGT                                                                                                                                                                                                                                                                                                               >  1:494935/1‑301`  
`ACCTTTGGTGGCGGTGAAGACGATTGGGCTGACGAAGTTGAAGAGAACGGCTATGTTGCCTCTGGTTCTGCCAAAGCGAGCAAACCACGCGACGAAGAAAGCTGGGACGAAGACGACGAAGAGTCCGAGGAAGCAGACGAAGACGGAGACTTCTAAGTGGAACTGCGGGAGAAAATCCTTGAGCGAATCAAGGTGACTTCCTCTGGGTGTTGGGAGTGGCAGGGCGCTACGAACAATAAAGGGTACGGGCAGGTGTGGTGCAGCAATACCGGAAAGGTTGTCTACTGTCATCGCGTAATGT                                                                                                                                                                                                                                                                                                               >  1:288488/1‑301`  
`ACCTTTGGTGGCGGTGAAGACGATTGGGCTGACGAAGTTGAAGAGAACGGCTATGTTGCCTCTGGTTCTGCCAAAGCGAGCAAACCACGCGACGAAGAAAGCTGGGACGAAGACGACGAAGAGTCCGAGGAAGCAGACGAAGACGGAGACTTCTAAGTGGAACTGCGGGAGAAAATCCTTGAGCGAATCAAGGTGACTTCCTCTGGGTGTTGGGAGTGGCAGGGCGCTACGAACAATAAAGGGTACGGGCAGGTGTGGTGCAGCAATACCGGAAAGGTTGTCTACTGTCATCGCGTAATGT                                                                                                                                                                                                                                                                                                               >  1:79370/1‑301`  
`ACCTTTGGTGGCGGTGAAGACGATTGGGCTGACGAAGTTGAAGAGAACGGCTATGTTGCCTCTGGTTCTGCCAAAGCGAGCAAACCACGCGACGAAGAAAGCTGGGACGAAGACGACGAAGAGTCCGAGGAAGCAGACGAAGACGGAGACTTCTAAGTGGAACTGCGGGAGAAAATCCTTGAGCGAATCAAGGTGACTTCCTCTGGGTGTTGGGAGTGGCAGGGCGCTACGAACAATAAAGGGTACGGGCAGGTGTGGTGCAGCAATACCGGAAAGGTTGTCTACTGTCATCGCGTAATGT                                                                                                                                                                                                                                                                                                               >  1:275664/1‑301`  
`ACCTTTGGTGGCGGTGAAGACGATTGGGCTGACGAAGTTGAAGAGAACGGCTATGTTGCCTCTGGTTCTGCCAAAGCGAGCAAACCACGCGACGAAGAAAGCTGGGACGAAGACGACGAAGAGTCCGAGGAAGCAGACGAAGACGGAGACTTCTAAGTGGAACTGCGGGAGAAAATCCTTGAGCGAATCAAGGTGACTTCATCTGGGTGTTGGGAGTGGCAGGGCGCTACGAACAATAAAGGGTACGGGCAGGTGTGGTGCAGCAATACCGGAAAGGTTGTCTACTGTCATCGCGTAATGT                                                                                                                                                                                                                                                                                                               >  1:380116/1‑301`  
`ACCTTTGGTGGCGGTGAAGACGATTGGGCTGACGAAGTTGAAGAGAACGGCTATGTTGCCTCTGGTTCTGCCAAAGCGAGCAAACCACGCGACGAAGAAAGCTGGGACGAAGACGACGAAGAGTCCGAGGAAGCAGAAGAAGACGGAGACTTCTAAGTGGAACTGCGGGAGAAAATCCTTGAGCGAATCAAGGTGACTTCCTCTGGGTGTTGGGAGTGGCAGGGCGCTACGAACAATAAAGGGTACGGGCAGGTGTGGTGCAGCAATACCGGAAAGGTTGTCTACTGTCATCGCGTAATGT                                                                                                                                                                                                                                                                                                               >  1:366399/1‑301`  
`ACCTTTGGTGGCGGTGAAGACGATTGGGCTGACGAAGTTGAAGAGAACGGCTATGTTGCCTCTGGTTCTGCCAAAGCGAGCAAACCACGCGACGAAGAAAGCTGGGACGAAGACGACGAAGAGTCAGAGGAAGCAGACGAAGACGGAGACTTCTAAGTGGAACTGCGGGAGAAAATCCTTGAGCGAATCAAGGTGACTTCCTCTGGGTGTGGGGAGTGGCAGGGCGCTACGAACAATAACGGGTACGGGCAGGTGTGGTGCAGCAATACCGGAAAGGTTGTCTACTGTCATCGCGTAATGT                                                                                                                                                                                                                                                                                                               >  1:226429/1‑301`  
`ACCTTTGGTGGCGGTGAAGACGATTGGGCTGACGAAGTTGAAGAGAACGGCTATGTTGCACCTGGTTCTGCCCAAGCTAGCAAACCACGCGCCGAAGAAAGCTGGGACGAAGACGACGAAGAGTCCGAGGAAGCAGACGAAGACGGAGACTTCTAAGTGGAACTGCGGGAGAAAATCCTTGAGCGAATCAAGGTGACGTCCTCTGGGTGTTGGGAGTGGCAGGGCGCTACGAACAATAAAGGGTACGGGCAGGTGTGGTGCAGCAATAACGGAAAGGTTGTCTAATGTGATCGCGTACTGT                                                                                                                                                                                                                                                                                                               >  1:261096/1‑301`  
`ACCTTTGGTGGCGGTGAAGACGATTGGGCTGACGAAGTTGAAGAGAACGGATATGTTGCCTCTGGTTCTGCCAAAGCGAGCAAACCACGCGACGAAGAAAGCTGGGACGAAGACGACGAAGAGTCCGAGGAAGCAGACGAAGACGGAGACTTCTAAGTGGAACTGCGGGAGAAAATCCTTGAGCGAATCAAGGTGACTTCCTCTGTGTGTTGGGAGTGGCAGGGCGCTACGAACAATAAAGGGTACGGGCAGGTGTGGTGCAGCAATACCGGAAAGGTTGTCTACTGTCATCGCGTAATGT                                                                                                                                                                                                                                                                                                               >  1:255289/1‑301`  
`ACCTTTGGGTGCGGTTAAAACGATTGGGGTGACGAAGTTGAAGGGAACGTCTATGTTGCCTCTGGTTCTGCCAAAGCGAGAAAACCACGCGACGAAGAAAGCTGGGACGAAGACGACGAAGAGTCCGAGGAAGCAGACGAAGACGGAGACTTCTAAGTGGAACTGCGGGAGAAAATCCTTGAGCGAATCAAGGTGACTTCCTCTGGGTGTTGGGAGTGGCAGGGCGCTACGAACAATAAAGGGTACGGGCAGGTGTGGTGCAGCAATACCGGAAAGGTTGTCTACTGTCATCGCGTAATGT                                                                                                                                                                                                                                                                                                               <  1:398985/301‑1`  
 `ccgTTGGTGGCGGTGAATCCGATTGTGCTGACGAAGTTGAAGAGAACGGCTATGTTGCCTCTGGTTCTGCCAAAGCGAGCAAACCACGCGACGAATAAAGCTGGGACGAAGAAGACGAAGAGTCCGAAGAATAAAACCAAGACGGAGACTTCTAAGGGGAACTGCGGGAGAAATTCCTTGGGCGAATCAAGGTGACTTCCTCTGGGTGTTGGGAGTGGCAGGGCGCTACGAACAATAAAGGGTACGGGCAGGTGTGGTGCAGCAATACCGGAAAGGTTGTCTACTGTCATCGCGTAATGTC                                                                                                                                                                                                                                                                                                              <  1:330136/298‑1`  
 `aCTTTGGTGGCGGTGAAGGCGATTGGGGTGACGAAGTTGAAGAGAACGGATATGTTGCCTCTGGTTCTGCCAAAGCGAGCAAACCACGCGACGAAGAAAGCTGGGACGAAGACGACGAAGAGTACGAGGAAGCAGACGAAGACGGAGACTGCTAAGTGGAACTGCGGGAGAAAATCCTTGAGCGAATCAAGGTGACTTCCTCTGGGTGTTGGGAGTGGCAGGGCGCTACGAACAATAAAGGGTACGGGCATGTGTGGTGCAGCAATACCGGAAAGGTTGTCTACTGTCATCGCGTAATGTC                                                                                                                                                                                                                                                                                                              <  1:4849/300‑1`  
 `CCTTTGGTGGCGGTGTAGACGATTGGGCTGACGAAGTTGAAGAGAACTGCTATGTTGCCTCTGGTTCTGCCAAAGCGAGCAAACCACGCGACGAAGAAAGCTGGGACGAAGACGACGAAGAGTCCGAGGAAGCAGACGAAGACGGAGACTTCTAAGTGTAACTGCGGGAGAAAATCCTTGAGCGAATCAAGGTGACTTCCTCTGGGTGTTGGGATTGGCAGGGCGCTACGAACAATAAAGGGTACGGGCAGGTGTGGGGCAGCAATACCGGAAAGGTTGTCTACTGTCATCGCGTAATGTC                                                                                                                                                                                                                                                                                                              >  1:9919/1‑301`  
 `CCTTTGGTGGCGGTGAAGACGATTGGGCTGCCGAAGTTGAAGAGAACGGCTATGTTGCCTCTGGTTCTGCCAAAGCGAGCAAACCACGCGACGAAGAAAGCTGGGACGAAGACTACGAAGAGTCCGCGGAAGCAGACGAAGACGGAGACTTCTAAGTGGAACTGCGGGAGAAAATCCTTGAGAGAATCAAGGTGACTTCCTCTGGGTGTTGGGAGTGGCAGGGCGCTACGAACAATAAAGGGTACGGGCAGGTGAGGTGCAGCAATACCGGAAAGGGTGTCTACTGTCAGCGCGTAATGTC                                                                                                                                                                                                                                                                                                              >  1:94644/1‑301`  
 `CCTTTGGTGGCGGTGAAGACGATTGGGCTGACGAAGTTTAAGAGAACGGCTATGTTGCCTCTGGTTCTGCCAAAGCGAGCAAACCACGCGACGAAGAAAGCTGGGACGAAGACGACGAAGAGTCCGAGGAAGCAGACGAAGACGGAGACTTCTAAGTGGAACTGCGGGAGAAAATCCTTGAGCGAATCAAGGTGACTTCCTCTGGGTGTTGGGAGTGGCAGGGCGCTACGAACAATAAAGGGTACGGGCAGGTGTGGTGCAGCAATACCGGAAAGGTTGTCTACTGTCATCGCGTAATGTC                                                                                                                                                                                                                                                                                                              >  1:452371/1‑301`  
 `CCTTTGGTGGCGGTGAAGACGATTGGGCTGACGAAGTTGAAGAGAACGGCTATGTTGCCTCTGGTTCTGCCAAAGTGAGCAAACCACGCGACGAAGAAAGCTGGGACGAAGACGACGAAGAGTCCGAGGAAGCAGACGAAGACGGAGACTTCTAAGTGGAACTGCGGGAGAAAATCCTTGAGCGAATCAAGGTGACTTCCTCTGGGTGTTGGGAGTGGCAGGGCGCTACGAACAATAAAGGGTACGGGCAGGTGTGGTGCAGCAATACCGGAAAGGTTGTCTACTGTCATCGCGTAATGTC                                                                                                                                                                                                                                                                                                              >  1:159357/1‑301`  
 `CCTTTGGTGGCGGTGAAGACGATTGGGCTGACGAAGTTGAAGAGAACGGCTATGTTGCCTCTGGTTCTGCCAAAGCGAGCAAACCACTCGACGAAGAAAGCTGGGACGAAGACGACGAAGAGTCCGAGGAAGCAGACGAAGACGGAGACTTCTAAGTGGAACTGCGGGAGAAAATCCTTGAGTGAATCAAGGTGACTTCCTCTGGGTGTTGGGAGTGGCAGGGCGCTACGAACAATAAAGGGTACGGGCAGGTGTGGTGCAGCAATACCGGAAAGGTTGTCTACTGTCATCGCGTAATGTC                                                                                                                                                                                                                                                                                                              >  1:122679/1‑301`  
 `CCTTTGGTGGCGGTGAAGACGATTGGGCTGACGAAGTTGAAGAGAACGGCTATGTTGCCTCTGGTTCTGCCAAAGCGAGCAAACCACGCGACGAAGAAAGCTGGGACGAAGACGACGAAGAGTCCGAGGAAGCAGACGACGACGGAGACTTCTAAGTTGAACTGCGGGAGAAAATCCTTGAGCGAATCAAGGTGACTTCCTCTGGGTGTTGGGAGTGGCAGGGCGCTACGAACAATAAAGGGTACGGGAAGGTGTGGTGGAGCACGACCGGAAAGGTTGTCTCCTGTCATCGCGTAATTTC                                                                                                                                                                                                                                                                                                              >  1:302528/1‑301`  
 `CCTTTGGTGGCGGTGAAGACGATTGGGCTGACGAAGTTGAAGAGAACGGCTATGTTGCCTCTGGTTCTGCCAAAGCGAGCAAACCACGCGACGAAGAAAGCTGGGACGAAGACGACGAAGAGTCCGAGGAAGCAGACGACGACGGAGACTTCTAAGTGGAACTGCGGGAGAAAATCCTTGAGCGAATCAAGGTGACATCCTCTGGGTGTTGGGAGTGGCAGGGCGCTACGAACAATAAAGGTTACGGGCAGGTGTGGTGAAGCAATACCGGAAAGGTTGTCTACTGTCATCGTGTAATGTt                                                                                                                                                                                                                                                                                                              >  1:258383/1‑300`  
 `CCTTTGGTGGCGGTGAAGACGATTGGGCTGACGAAGTTGAAGAGAACGGCTATGTTGCCTCTGGTTCTGCCAAAGCGAGCAAACCACGCGACGAAGAAAGCTGGGACGAAGACGACGAAGAGTCCGAGGAAGCAGACGAAGACGGAGACTTCTAAGTGGAACTTCGGGAGAAAATCCTTGAGCGAATCAAGGTGACTTCCTCTGGGTGTTGGGAGTGGCAGGGCGCTACGAACAATAAAGGGTACGGGCAGGTGTGGTGCAGCAATACCGGAAAGGTTGTCTACTGTCATCGCGTAATGTC                                                                                                                                                                                                                                                                                                              >  1:376755/1‑301`  
 `CCTTTGGTGGCGGTGAAGACGATTGGGCTGACGAAGTTGAAGAGAACGGCTATGTTGCCTCTGGTTCTGCCAAAGCGAGCAAACCACGCGACGAAGAAAGCTGGGACGAAGACGACGAAGAGTCCGAGGAAGCAGACGAAGACGGAGACTTCTAAGTGGAACTGCGGGAGAATATCCTTGAGCGAATCAAGGTGACTTCCTCTGGGTGTTGGGAGTGGCAGGGCGCTACGAACAATAAAGGGTACGGGCAGGTGTGGTGCAGCAATACCGGAAAGGTTGTCTACTGTCATCGCGTAATGTC                                                                                                                                                                                                                                                                                                              >  1:179301/1‑301`  
 `CCTTTGGTGGCGGTGAAGACGATTGGGCTGACGAAGTTGAAGAGAACGGCTATGTTGCCTCTGGTTCTGCCAAAGCGAGCAAACCACGCGACGAAGAAAGCTGGGACGAAGACGACGAAGAGTCCGAGGAAGCAGACGAAGACGGAGACTTCTAAGTGGAACTGCGGGAGAAAATCCTTTAGCGAATCAAGGTGACTTCCTCTGGGTGTTGGGAGTGGCAGGGCGCTACGAACAATAAAGGGTACGGGCAGGTGTGGTGCAGCAATACCGGAAAGGTTGTCTACTGTCATCGCGTAATGTC                                                                                                                                                                                                                                                                                                              >  1:69740/1‑301`  
 `CCTTTGGTGGCGGTGAAGACGATTGGGCTGACGAAGTTGAAGAGAACGGCTATGTTGCCTCTGGTTCTGCCAAAGCGAGCAAACCACGCGACGAAGAAAGCTGGGACGAAGACGACGAAGAGTCCGAGGAAGCAGACGAAGACGGAGACTTCTAAGTGGAACTGCGGGAGAAAATCCTTGAGCGAATCAAGGTGACTTCCTCTGGGTGTTGGGAGTGGTAGGGCGCTACGAACAATAAAGGGTACGGGCATGTGTGGTGCAGCAATACCGGAAAGGTTGTCTACTGTCATCGCGTAATGgt                                                                                                                                                                                                                                                                                                              >  1:509519/1‑299`  
 `CCTTTGGTGGCGGTGAAGACGATTGGGCTGACGAAGTTGAAGAGAACGGCTATGTTGCCTCTGGTTCTGCCAAAGCGAGCAAACCACGCGACGAAGAAAGCTGGGACGAAGACGACGAAGAGTCCGAGGAAGCAGACGAAGACGGAGACTTCTAAGTGGAACTGCGGGAGAAAATCCTTGAGCGAATCAAGGTGACTTCCTCTGGGTGTTGGGAGTGGCAGGGCGCTACGAACAATAAAGGGTACGGGCAGGTGTGGTGCAGCAATGCCGGAAAGGTTGTCTACTGTCATCGCGTAATGTC                                                                                                                                                                                                                                                                                                              >  1:253005/1‑301`  
 `CCTTTGGTGGCGGTGAAGACGATTGGGCTGACGAAGTTGAAGAGAACGGCTATGTTGCCTCTGGTTCTGCCAAAGCGAGCAAACCACGCGACGAAGAAAGCTGGGACGAAGACGACGAAGAGTCCGAGGAAGCAGACGAAGACGGAGACTTCTAAGTGGAACTGCGGGAGAAAATCCTTGAGCGAATCAAGGTGACTTCCTCTGGGTGTTGGGAGTGGCAGGGCGCTACGAACAATAAAGGGTACGGGCAGGTGTGGTGCAGCAATACCGGTACGGTTGACTACTGTCATCGCTTAATGTC                                                                                                                                                                                                                                                                                                              >  1:109418/1‑301`  
 `CCTTTGGTGGCGGTGAAGACGATTGGGCTGACGAAGTTGAAGAGAACGGCTATGTTGCCTCTGGTTCTGCCAAAGCGAGCAAACCACGCGACGAAGAAAGCTGGGACGAAGACGACGAAGAGTCCGAGGAAGCAGACGAAGACGGAGACTTCTAAGTGGAACTGCGGGAGAAAATCCTTGAGCGAATCAAGGTGACTTCCTCTGGGTGTTGGGAGTGGCAGGGCGCTACGAACAATAAAGGGTACGGGCAGGTGTGGTGCAGCAATACCGGAAAGGTTGTCTACTGTCATCGCGTAATGTC                                                                                                                                                                                                                                                                                                              >  1:316691/1‑301`  
 `CCTTTGGTGGCGGTGAAGACGATTGGGCTGACGAAGTTGAAGAGAACGGCTATGTTGCCTCTGGTTCTGCCAAAGCGAGCAAACCACGCGACGAAGAAAGCTGGGACGAAGACGACGAAGAGTCCGAGGAAGCAGACGAAGACGGAGACTTCTAAGTGGAACTGCGGGAGAAAATCCTTGAGCGAATCAAGGTGACTTCCTCTGGGTGTTGGGAGTGGCAGGGCGCTACGAACAATAAAGGGTACGGGCAGGTGTGGTGCAGCAATACCGGAAAGGTTGTCTACTGTCATCGCGTAATGTC                                                                                                                                                                                                                                                                                                              >  1:141598/1‑301`  
 `CCTTTGGTGGCGGTGAAGACGATTGGGCTGACGAAGTTGAAGAGAACGGCTATGTTGCCTCTGGTTCTGCCAAAGCGAGCAAACCACGCGACGAAGAAAGCTGGGACGAAGACGACGAAGAGTCCGAGGAAGCAGACGAAGACGGAGACTTCTAAGTGGAACTGCGGGAGAAAATCCTTGAGCGAATCAAGGTGACTTCCTCTGGGTGTTGGGAGTGGCAGGGCGCTACGAACAATAAAGGGTACGGGCAGGTGTGGTGCAGCAATACCGGAAAGGTTGTCTACTGTCATCGAGTAATGTC                                                                                                                                                                                                                                                                                                              >  1:74890/1‑301`  
 `CCTTTGGTGGCGGTGAAGACGATTGGGCTGACGAAGTTGAAGAGAACGGCTATGTTGCCTCTGGTTCTGCCAAAGCGAGCAAACCACGCGACGAAGAAAGCTGGGACGAAGACGACGAAGAGTCCGAGGAAGCAGACGAAGACGGAGACTTCTAAGTGGAACTGCGGGAGAAAATCCTTGAGCGAATCAAGGTGACTTCCTCTGGGTGTTGGGAGTGGCAGGGCGCTACGAACAATAAAGGGTACGGGCAGGTGGGGTGCAGCAATACCGGAAAGGTTGGCTACTGTCATCGCGTAATGTg                                                                                                                                                                                                                                                                                                              >  1:314488/1‑300`  
 `CCTTTGGTGGCGGTGAAGACGATTGGGCTGACGAAGTTGAAGAGAACGGCTATGTTGCCTCTGGTTCTGCCAAAGCGAGCAAACCACGCGACGAAGAAAGCTGGGACGAAGACGACGAAGAGTCCGAGGAAGCAGACGAAGACGGAGACTTCTAAGTGGAACTGCGGGAGAAAATCCTTGAGCGAATCAAGGTGACTTCCTCTGGGTGTTGGGAGTGGCAGGGCGCTACGAACAATAAAGGGTACGGGAAGGTGTGGTGCAGCAATACCGGAAAGGTTGTCTACTGTCATCGCGTAATGTt                                                                                                                                                                                                                                                                                                              >  1:100387/1‑300`  
 `CCTTTGGTGGCGGTGAAGACGATTGGGCTGACGAAGTTGAAGAGAACGGCTATGTTGCCTCTGGTTCTGCCAAAGCGAACAAACCACGCGACGAAGAAAGCTGGGACGAAGACGACGAAGAGTCCGAGGAAGCAGACGAAGACGGAGACTTCTAAGTGGAACTGCGGGAGAAAATCCTTGAGCGAATCAAGGTGACTTCCTCTGGGTGGTGGGAGTGGCAGGGCGCTACGAACAATAAAGGGTACGGGCAGGTGTGGTGCAGCAATACCGGAAAGGTTGTCTACTGTCATCGCGTAATGTC                                                                                                                                                                                                                                                                                                              >  1:379015/1‑301`  
 `CCTTTGGTGGCGGTGAAGACGATTGGGCTGACGAAGTTGAAGAGAAAGGCTATGTTGCCTCTGGTTCTGCCAAAGCGAGCAAACCACGCGACGAAGAAAGCGGGGACGAAGACGACGAAGAGTCCGAGGAAGCAGACGAAGACGGAGACTTCTAAGTGGAACTGCGGGAGAAAATCCTTCAGCGAATCAAGGTGACTTCCTCTGGGTGTTGGGATTGGCAGGGCTCTACGAACAATCAACGGTACGGGCCGGGGTGGTTCAGCAATACCGGACAGGTTGTCTACTGTCATCTCGTAATGct                                                                                                                                                                                                                                                                                                              >  1:283777/1‑299`  
 `CCTTTGGTGGCGGTGAAGACGATTGGGATGACGAAGTTGAAGAGAACGGCTATGTTGCCTCTGGTTCTGCCAAAGCGAGCAAACCACGCGACGAAGAAAGCTGGGACGAAGACGACGAAGAGTCCGAGGAAGCAGACGAAGACGGAGACTTCTAAGTGGAACTGCGGTAGAAAATCCTTGAGCGAATCAAGGTGACTTCCTCTGGGTGTTGGGAGTGGCAGGGCGCTACGAACAATAAAGGGTACGGGCAGGTGTGGTGCAGCAATACCGGAAAGGTTGTCTACTGTCATCGCGTAATGTt                                                                                                                                                                                                                                                                                                              >  1:357495/1‑300`  
 `CCTTGGGTGGCGGTGAAGACGATTGGGCTGACGAAGTTGAAGAGAACGGCTATGTTGCCTCTGGTTCTGCCAAAGCGAGCAAACCACGAGACGAAGAAAGCTGGGACGAAGACGACGAAGAGTCCGAGGAAGCAGACGAAGACGGAGACTTCTAAGTGGAACTGCGGGAGAAAATCCTTGAGCGAATCAAGGTGACTTCCTCTGGGTGTTGGGAGTGGCAGGGCGCTACGAACAATAAAGGGTACGGGCAGGTGTGGTGCAGCAATACCGGAAAGGTTGTCTACTGTCATCGCGTAATGTC                                                                                                                                                                                                                                                                                                              <  1:173326/301‑1`  
            `GGTGAAGACGATTGGGCTGACGAAGTTGAAGAGAACGGCTATGTTGCCTCTGGTTCTGCCAAAGCGAGCAAACCACGCGACGAAGAAAGCTGGGACGAAGACAACGAAGAGTCTGAGGAAGCAGACGAAGACGGAGACTTCTAAGTGGAACTGCGGGAGAAAATCCTTGAGCGAATCAAGGTGACTTCCTCTGGGTGTTGGGAGTGGCAGGGCGCTACGAACAATAAAGGGTACGGGCAGGTGTGGTGCAGCAATACCGGAAAGGTTGTCTACTGTCATCGCGTAATGTCCtgtctcttat                                                                                                                                                                                                                                                                                                   >  1:133474/1‑291`  
            `GGTGAAGACGATTGGGCTGACGAAGTTGAAGAGAACGGCTATGTTGCCTATGGTTCTGCCAAAGCGAGCAAACCACGCGACGAAGAAAGCTGGGACGAAGACGACGAAGAGTCCGAGGAAGCAGACGAAGACGGAGACTTCTAAGTGGAACTGCGGGAGAAAATCCTTGAGCGAATCAAGGTGACTTCCTCTGGGTGTTGGGAGTGGCAGGGCGCTACGAACAATAAAGGGTACGGGCAGGTGTGGTGCAGCAATACCGGAAAGGTTGTCTACTGTCATCGCGTAATGctgtctcttatac                                                                                                                                                                                                                                                                                                   >  1:348870/1‑288`  
                    `CGATTGGGCTGACGAAGTTGAAGAGAACGGCTATGTTGCCTCTGGTTCTGCCAAAGCGAGCAAACCACGCGACGAAGAAAGCTGGGACGAAGACGACGAAGAGTCCGAGGAAGCAGACGAAGACGGAGACTTCTAAGTGGAACTGCGGGAGAAAATCCTTGAGCGAATCAAGGTGACTTCCTCTGGGTGTTGGGAGTGGCAGGGCGCTACGAACAATAAAGGGTACGGGCAGGTGTGGTGCAGCAATACCGGAAAGGTTGTCTACTGTCATCGAGTAATGctgtctcttatacacatctcc                                                                                                                                                                                                                                                                                           >  1:298911/1‑280`  
                                                     `TGTTGCCTCTGGTTCTGCCAAAGCGAGCAAACCACGCGACGAAGAAAGCTGGGACGAAGACGACGAAGAGTCCGAGGAAGCAGACGAAGACGGAGACTTCTAAGTGGAACTGCGGGAGAAAATCCTTGAGCGAATCAAGGTGACTTCCTCTGGGTGTTGGGAGTGGCAGGGCGCTACGAACAATAAAGGGTACGGGCAGGTGTGGTGCAGCAATACCGGAAAGGTTGTCTACTGTCATCGCGTAATGTCCAATGTATCCTTAGCTCGCACCTATGGTGTCTCCCAACAGACTATTTGTGAT                                                                                                                                                                                                                                                          <  1:53911/301‑1`  
                                                         `GCCTCTGGTTCTGCCAAAGCGAGCAAACCACGCGACGAAGAAAGCTGGGACGAAGACGACGAAGAGTCCGAGGAAGCAGACGAAGACGGAGACTTCTAAGTGGAACTGCGGGAGAAAATCCTTGAGCGAATCAAGGTGACTTCCTCTGGGTGTTGGGAGTGGCAGGGCGCTACGAACAATAAAGGGTACGGGCAGGTGTGGTGCAGCAATACCGGAAAGGTTGTCTACTGTCATCGCGTAATGTCCAATGTATCCTTAGCTCGCACCTATGGTGTCTCCCAACAGACTATTTGTGATATAC                                                                                                                                                                                                                                                      <  1:4988/301‑1`  
                                                                                             `GAAGAAAGCTGGGACGAAGACGACGAAGAGTCCGAGGAAGCAGACGAAGACGGAGACTTCTAAGTGGAACTGCGGGAGAAAATCCTTGAGCGAATCAAGGTGACTTCCTCTGGGTGTTGGGAGTGGAAGGGCGCTACGAACAATAAAGGGTACGGGCAGGTGTGGTGCAGCAATACCGGAAAGGTTGTCTACTGTCATCGCGTAATGTCCAATGTATCCTTAGCTCGCACCTATGGTGTCTCCAAACAGACTATTTGTGATATACGCAAAGGGAGGCGACATGGCAGGTTACGACCCTAAA                                                                                                                                                                                                                  >  1:255484/1‑301`  
                                                                                                                                         `CGAAGACGGAGACTTCTAAGTGGAACTGCGGGAGAAAATCCTTGAGCGAATCAAGGTGACTTCCTCTGGGTGTTGGGAGTGGCAGGGCGCTACGAACAATAAAGGGTACGGGCAGGTGTGGTGCAGCAATACCGGAAAGGTTGTCTACTGTCATCGCGTAATGTCCAATGTATCCTTAGCTCGCACCTATGGTTTCGCCCAACCGACTATTTGTGATATACGCCAAGGGAGGCTACATGGCAGGTTACGGCGCTAAAGGAATCCGCAAGGTTGGAGCGTTTAGCTCTGGCCTAGAGGACAA                                                                                                                                                                      >  1:282157/1‑301`  
                                                                                                                                                              `GGAAATGCGGGAGAAATGCCTTGGTCGAATCAAGGTGACTTCCGTTGGGTGTTGGTAGTGGAAGGGCGCTACGAACAATAAAGGGTACGGGCAGGTGTGGTGCAGCAATACCGGAAAGGTTGTCTACTGTCATCGCGTAATATCCAATGTATCCTTAGCTGGCACCTATGGTGTCTCCCAACAGACTATTTGTGATATACGCAAAGGGAGGCGACATGGCAGGTTACGGTGCTAAAGGAATCCGAAAGGTTGGAGCGTTTCGCTCTGGCCTAGAGGACAAGGTTTCAAAGCAGTTGGAATC                                                                                                                                                 <  1:41140/301‑1`  
                                                                                                                                                                             `AATCCTTGGGCGTATCAAGGGGACTTCCTCGGGGTGTTGGGGGTGGCAGGGCGCTACGAACAATAAAGGGTACGGGCAGGTGTGGTGCAGCAATACCGGAAAGGTTGTCTACTGTCATCGCGTAATGTCCAATGTATCCTTAGCTCGCACCTATGGTGTCTCCCAACAGACTATTTGTGATATACGCAAAGGGAGGCGACATGGCAGGTTACGGCGCTAAAGGAATCCGAAAGGTTGGAGCGTTTCGCTCTGGCCTAGAGGACAAGGTTTCAAAGCAGTTGGAATCAAAAGGTATTAAATT                                                                                                                                  <  1:10241/301‑1`  
                                                                                                                                                                                               `agaaaagTCCTCTGGGTGGTGGGAGTGGCGGGGCGCTATGAACAAGAAAGGGTACGGGCAGTTGTTGTGAAGCAATACCAGAAAGGTTGTCTACTGTCATCGCGGAATGTCAAATGTATCCTTAGCACGCACCTATGGTGACTCCCAACAGACTATTTGTGATATACGCAAAGGGAGGCGACATGGCAGGTGACGGCGATAAAGGAATCCGAAAGGTTGGAGCGTTTCGCTCTGCCCTAGAGGACAAGGTTTCAAAGCAGTTGGAATCAAAAGGTATTAAATTCGAGTATGAAGAGTGGAA                                                                                                                <  1:46197/294‑1`  
                                                                                                                                                                                                       `CCTCTGGGTGTTGGGAGTGGCAGGGCGCTACGAACAATAAAGGGTACGGGCAGGTGTGGTGCAGCAATACCGGAAAGGTTGTCTACTGTCATCGCGTAATGTCCAATGTATCCTTAGCTCGCACCTATGGTGTCTCCCAACAGACTATTTGTGATATACGCAAAGGGAGGCGACCTGGCAGGTTACGGCGCTAAAGGAATCCGAAAGGTTGGAGCGTTTCGCTCTGGCCTAGAGGACAAGGTTTCAAAGCAGTTTGAATCAAAAGGTATTAAATTCGAGTATGAAGAGTGGAAAGTGCCTT                                                                                                        >  1:414467/1‑301`  
                                                                                                                                                                                                                                         `CAATAAAGGGTACGGGCAGGTGTGGTGCAGCAATACCGGAAAGGTTGTCTACTGTCATCGCGTAATGTCCAATGTATCCTTAGCTCGCACCTATGGTGTCTCCCAACAGACTATTTGTGATATACGCAAAGGGAGGTGACATGGCAGGTTACGGCGCTAAAGGAATCCGAAAGGTTGGAGCGTTTCGCTCTGGCCTAGAGGACAAGGTTTTAAAGCAGTTGGAATCAAAAGGTATTAAATTCGAGTATGAAGAGTGGAAAGTGCCTTATGTAATTCCtgtctcttatacacatctccgagc                                                                      >  1:281961/1‑277`  
                                                                                                                                                                                                                                                           `GGTGTGGTGCAGCAATACCGGAAAGGTTGTCTACTGTCATCGCGTAATGTCCAATGTATCCTTAGCTCGCACCTATGGTGTCTCCCAACAGACTATTTGTGATATACGCAAAGGGAGGCGACATGGCAGGTTACGGCGCTAAAGGAATCCGAAAGGTTGGAGCGTTTCGCTCTGGCCTAGAGGACAAGGTTTCAAAGCAGTTGGAATCAAAAGGTATTAAATTCGAGTATGAAGAGTGGAACGTGCCTTATGTAATTCCGGCGAGCAATCACACTTACACTCCAGACTTCTTCCTTCCAAA                                                    >  1:199159/1‑301`  
                                                                                                                                                                                                                                                                               `GAAAGGTTGTCTACTGTCATCGCGTAATGTCCAATGTATCCTTAGCTCGAACCTATGGTGTCTCCCAACAGACTATTTGTGATATACGCAAAGGGAGGCGACATGGCAGGTTACGGCGCTAAAGGAATCCGAAAGGTTGGAGCGTTTCGCTCTGGCCTAGAGGACAAGGTTTCAAAGCAGTTGGAATCAAAAGGTATTAAATTCGAGTATGAAGAGTGGAAAGTGCCTTATGTAATTCCGGCGAGCAATCACACTTACACTCCAGACTTCTTACTTCCAAACGGTATATTCGTTGAGACAA                                >  1:438896/1‑301`  
                                                                                                                                                                                                                                                                                       `GTCTACTGTCATCGCGTAATGTCCAATGTATCCTTAGCTCGCACCTATGGTGTCTCCCAACAGACTATTTGTGATATACGCAAAGGGAGGCGACATGGCAGGTTACTGCGCTAAAGGAATCCGAAAGGTTGGAGCGTTTCGCTCTGGCCTAGATGACAAGGTTTCAAAGCAGTTGGAATCAAAAGGTATTAAATTCGAGTATGAAGAGTGGAAAGTGCCTTATGTAATTCCGGCGAGCAATCACACTTACACTCCAGACTTCTTACTTCCAAACGGTATATTCGTTGAGACAAAGGGTCTG                        >  1:459689/1‑301`  
                                                                                                                                                                                                                                                                                                  `gtccacggaggCCAATTTATCATTAGCTCGCACCTATGGTTTCTCCCAACAGACTATTTGTGATATACGCAAAGGGAGGCGACATGGCAGGTTACGGCGCTAAAGGAATCCGAAAGGTTGGAGCGTTTCGCTCTGGCCTAGAGGACAAGGTTTCAAAGCAGTTGGAATCAAAAGGTATTAAATTCGAGTATGAAGAGTGGAAAGTGCCTTATGTAATTCCGGCGAGCAATCACACTTACACTCCAGACTTCTTACTTCCAAACGGTATATTCGTTGAGACAAAGGGTCTGTGGGAAAGCGA             <  1:185499/290‑1`  
                                                                                                                                                                                                                                                                                                        `cagGTCCAATGTATCATTAGCTCGCACCTATGGTGTCTCCCAACAGACTATTTGTGATATACGCAAAGGGAGGCGACATGGCAGGTTACGGCGCTAAAGGAATCCGAAAGGTTGGAGCGTTTCGCTCTGGCCTAGAGGACAAGGTTTCAAAGCAGTTGGAATCAAAAGGTATTAAATTCGAGTATGAAGAGTGGAAAGTGCCTTATGTAATTCCGGCGAGCAATCACACTTACACTCCAGACTTCTTACTTCCAAACGGTATATTCGTTGAGACAAAGGGTCTGTGGGAAAGCGATGATAG       <  1:361123/298‑1`  
                                                                                                                                                                                                                                                                                                          `atTCCAATGAGTCCTTAGCTCGCCTCTATGGTGACTCCCAACAGACTATTTGTGATATACGCAAAGGGAGGCAACATGGCAGGTTACGGCGCTAAAGGAATCCGAAAGGTGGGAGCGTTTCGCTCTGTCCTAGAGGACAAGGTTTCAAAGCAGTTGGAATCAAAAGGTATTAATTTCGAGTATGAAGAGTGGAAAGTGCCTTATGTAATTCCGGCGAGCAATCACACTTACACTCCAGACTTCTTACTTCCAAACGGTATATTCGTTGAGACAAAGGGTCTGTGGGAAAGAGATGATAGAA     <  1:94901/299‑1`  
                                                                                                                                                                                                                                                                                                           `GTCCAATGTTTCCTTAGCTCGCACCTATGGTGTCTCCCAACAGACTATTTGTGATATACGCAAAGGGAGGCGACATGGCAGGTTACGGCGCTAAAGGAATCCGAAAGGTTGGAGCGTTTCGCTCTGGCCTAGAGGACAAGGTTTCAAAGCAGTTGGAATCAAAAGGTATTAAATTCGAGTATGAAGAGTGGAAAGTGCCTTATGTAATTCCGGCGAGCAATCACACTTACACTCCAGACTTCTTACTTCCAAACGGTATATTCGTTGAGACAAAGGGTCTGTGGGAAAGCGATGATAGctt    >  1:42711/1‑298`  
                                                                                                                                                                                                                                                                                                           `GTCCAATGTATCCTTAGCTCGCACCTATGGTGTCTCCCAACAGTCTATTTGTGATATACGCAAAGGGAGGCGACATGGCAGGTTACGGCGCTAAAGGAATCCGAAAGGTTGGAGCGTTTCGCTCTGGCCTAGAGGACAAGGTTTCAAAGCAGTTGGAATCAAAAGGTATTAAATTCGAGTATGAAGAGTGGAAAGTGTCTTATGTAATTCCGGCGAGCAATCACACTTACACTCCAGACTTCTTACTTCCAAACGGTATATTCGTTGAGACAAAGGGTCTGTGGGAAAGCGATGATAGAAc    >  1:330442/1‑300`  
                                                                                                                                                                                                                                                                                                           `GTCCAATGTATCCTTAGCTCGCACCTATGGTGTCTCCCAACAGACTATTTGTGATATATGCAAAGGGAGGCGACATGGCAGGTTACGGCGCTAAAGGAATCCGAAAGGTTGGAGCGTTTCGCTCTGGCCTAGAGGACAAGGTTTCAAAGCAGTTGGAATCAAAAGGTATTAAATTCGAGTATGAAGAGTGGAAAGTGCCTTATGTCATTCCGGCGAGCAATCACACTTACACTCCAGACTTCTTACTTCCAAACGGTCTATTCGTTGAGCCAAAGGcctttctcttatacacatctccgag    >  1:176141/1‑276`  
                                                                                                                                                                                                                                                                                                           `GTCCAATGTATCCTTAGCTCGCACCTATGGTGTCTCCCAACAGACTATTTGTGATATACTCAAAGGGAGGCGACATGGCAGGTTACGGCGCTAAAGGAATCCGAAAGGTTGGAGCGTTTCGCTCTGGCCTAGAGGACAAGGTTTCAAAGCAGTTGGAATCAAAAGGTATTAAATTCGAGTATGAAGAGTGGAAAGTGCCTTATGTAATTCCGGCGAGCAATCACACTTACACTCCAGACTTCTTACTTCCAAACGGTATATTCGTTGAGACAAAGGGTCTGTGGGAAAGCGATGATAGAAA    >  1:371715/1‑301`  
                                                                                                                                                                                                                                                                                                           `GTCCAATGTATCCTTAGCTCGCACCTATGGTGTCTCCCAACAGACTATTTGTGATATACGCAAAGGTAGGCGACATGGCAGGTTACGGCGCTAAAGGAATCCGAAAGGTTTGAGCGTTTCGCTGTGGCCTAAAGGACAAGGTTTCAAAGCAGTTGGAATCAAAAGGTATTAAATTCGAGTATGAAGAGTGGAAAGTGCCTTATGTAATTCCGGCGAGCAATCACACTTACACTCCAGACTTCTTACTTCCAAACGGTATATTCGTTGAGACAAAGGGTCTGTGGGAAAGCGATGATAGAAA    >  1:448346/1‑301`  
                                                                                                                                                                                                                                                                                                           `GTCCAATGTATCCTTAGCTCGCACCTATGGTGTCTCCCAACAGACTATTTGTGATATACGCAAAGGGCGGCGACATGGCAGGTTACGGCGCTAAAGGAATCCGAAAGGTTGGAGCGTTTCGCTCTGGCCTAGAGGACACGGTTTCAAAGCAGTTGGAATCAAAAGGTATTAAATTCGAGTATGAAGAGTGGAAAGCGCCTTATGTAATTCCGGCGAGCAATCACACTTACACTCCAGACTTCTTACTTCCAAACGGTATATTCGTTGAGACAAAGGGTCctgtctcctatacacatctccg    >  1:484684/1‑279`  
                                                                                                                                                                                                                                                                                                           `GTCCAATGTATCCTTAGCTCGCACCTATGGTGTCTCCCAACAGACTATTTGTGATATACGCAAAGGGAGGCTACATGGCAGGTTACGGCGCTAAATGAATCCGAAAGGTTGGAGCGTTTCGCTCTGGCCTAGAGGACAAGGTTTCAAAGCAGTTGGAATCAAAAGGTATTAAATTCGAGTATGAAGAGTGGAAAGTGCCTTATGTAATTCCGGCGAGCAATCACACTTACACTCCAGACTTCTTACTTCCAAACGGTATAATCGTTGAGACAAAGGGTCTGTGGGAAAGCGATGATAGAAA    >  1:249601/1‑301`  
                                                                                                                                                                                                                                                                                                           `GTCCAATGTATCCTTAGCTCGCACCTATGGTGTCTCCCAACAGACTATTTGTGATATACGCAAAGGGAGGCGACATGGCAGGTTACGGCGCTAAAGTAATCCGAAAGGTTGGAGCGTTTCGCTCTGGCCTAGAGGACAAGGTTTCAAAGCAGTTGGAATCAAAAGGTATTAAATTCGAGTATGAAGAGTGGAAAGTGCCTTATGTAATTCCGGCGAGCAATCACACTGACACTCCAGACTTCTTACTTCCAAACGGTATATTCGTTGAGACAAAGGGTCTGTGGGCAAGCctgtctcttat    >  1:423221/1‑290`  
                                                                                                                                                                                                                                                                                                           `GTCCAATGTATCCTTAGCTCGCACCTATGGTGTCTCCCAACAGACTATTTGTGATATACGCAAAGGGAGGCGACATGGCAGGTTACGGCGCTAAAGGAATCCGAAAGGTTGGAGCGTTTCGCTCTGGCCTAGAGGACAATGTTTCAAAGCAGTTGGAATCAAAAGGTATTAAATTCGAGTATGAAGAGTGGAAAGTGCCTTATGTAATTCCGGCGAGCAATCACACTTACACTCCAGACTTCTTACTTCCAAACGGTATATTCGTTGAGACAAAGGGTCTGTGGGAAAGCGATGATAGAAA    >  1:100578/1‑301`  
                                                                                                                                                                                                                                                                                                           `GTCCAATGTATCCTTAGCTCGCACCTATGGTGTCTCCCAACAGACTATTTGTGATATACGCAAAGGGAGGCGACATGGCAGGTTACGGCGCTAAAGGAATCCGAAAGGTTGGAGCGTTTCGCTCTGGCCTAGAGGACAAGGTTTCAAAGCAGTTGGAATCAAAAGGTATTAAATTCGAGTATGAAGAGTGGAAAGTGCCTTATGTAATTCCGGCGATCAATCACACTTACACTCCAGACTTCTTACTTCCAAACGGTATATTCGTTGAGACAAAGGGTCTGTGGGAAAGCGATGATAGAAA    >  1:509042/1‑301`  
                                                                                                                                                                                                                                                                                                           `GTCCAATGTATCCTTAGCTCGCACCTATGGTGTCTCCCAACAGACTATTTGTGATATACGCAAAGGGAGGCGACATGGCAGGTTACGGCGCTAAAGGAATCCGAAAGGTTGGAGCGTTTCGCTCTGGCCTAGAGGACAAGGTTTCAAAGCAGTTGGAATCAAAAGGTATTAAATTCGAGTATGAAGAGTGGAAAGTGCCTTATGTAATTCCGGCGAGCACTCACACTTACACTCCAGACTTCTTACTTCCAACCGGTATATTCGTTGAGACAAAGGGTCTGTGGGAAAGCGATGATAGAAA    >  1:43484/1‑301`  
                                                                                                                                                                                                                                                                                                           `GTCCAATGTATCCTTAGCTCGCACCTATGGTGTCTCCCAACAGACTATTTGTGATATACGCAAAGGGAGGCGACATGGCAGGTTACGGCGCTAAAGGAATCCGAAAGGTTGGAGCGTTTCGCTCTGGCCTAGAGGACAAGGTTTCAAAGCAGTTGGAATCAAAAGGTATTAAATTCGAGTATGAAGAGTGGAAAGTGCCTTATGTAATTCCGGCGAGCAATCACACTTACTCTCCAGACTTCTTACTTCCAAACGGTATATTCGTTGAGACAAAGGGTCTGTGGGAAAGCGATGATAGAAA    >  1:102371/1‑301`  
                                                                                                                                                                                                                                                                                                           `GTCCAATGTATCCTTAGCTCGCACCTATGGTGTCTCCCAACAGACTATTTGTGATATACGCAAAGGGAGGCGACATGGCAGGTTACGGCGCTAAAGGAATCCGAAAGGTTGGAGCGTTTCGCTCTGGCCTAGAGGACAAGGTTTCAAAGCAGTTGGAATCAAAAGGTATTAAATTCGAGTATGAAGAGTGGAAAGTGCCTTATGTAATTCCGGCGAGCAATCACACTTACACTCCATACTTCTTACTTCCAAACGGTATATTCGTTGAGACAAAGGGTCTGTGGGAAAGCGATGATAGAAA    >  1:111926/1‑301`  
                                                                                                                                                                                                                                                                                                           `GTCCAATGTATCCTTAGCTCGCACCTATGGTGTCTCCCAACAGACTATTTGTGATATACGCAAAGGGAGGCGACATGGCAGGTTACGGCGCTAAAGGAATCCGAAAGGTTGGAGCGTTTCGCTCTGGCCTAGAGGACAAGGTTTCAAAGCAGTTGGAATCAAAAGGTATTAAATTCGAGTATGAAGAGTGGAAAGTGCCTTATGTAATTCCGGCGAGCAATCACACTTACACTCCAGACTTCTTACTTCCAAACGGTATATTCGTTGCTACAAAGGGTCTGTGGGAAAGCGATGATGGAAA    >  1:282268/1‑301`  
                                                                                                                                                                                                                                                                                                           `GTCCAATGTATCCTTAGCTCGCACCTATGGTGTCTCCCAACAGACTATTTGTGATATACGCAAAGGGAGGCGACATGGCAGGTTACGGCGCTAAAGGAATCCGAAAGGTTGGAGCGTTTCGCTCTGGCCTAGAGGACAAGGTTTCAAAGCAGTTGGAATCAAAAGGTATTAAATTCGAGTATGAAGAGTGGAAAGTGCCTTATGTAATTCCGGCGAGCAATCACACTTACACTCCAGACTTCTTACTTCCAAACGGTATATTCGTTGAGACAACGGGTCTGTGGGAActgtctcttataca    >  1:492170/1‑287`  
                                                                                                                                                                                                                                                                                                           `GTCCAATGTATCCTTAGCTCGCACCTATGGTGTCTCCCAACAGACTATTTGTGATATACGCAAAGGGAGGCGACATGGCAGGTTACGGCGCTAAAGGAATCCGAAAGGTTGGAGCGTTTCGCTCTGGCCTAGAGGACAAGGTTTCAAAGCAGTTGGAATCAAAAGGTATTAAATTCGAGTATGAAGAGTGGAAAGTGCCTTATGTAATTCCGGCGAGCAATCACACTTACACTCCAGACTTCTTACTTCCAAACGGTATATTCGTTGAGACAAAGGGTCTGctgtctcttatacacatctc    >  1:455937/1‑281`  
                                                                                                                                                                                                                                                                                                           `GTCCAATGTATCCTTAGCTCGCACCTATGGTGTCTCCCAACAGACTATTTGTGATATACGCAAAGGGAGGCGACATGGCAGGTTACGGCGCTAAAGGAATCCGAAAGGTTGGAGCGTTTCGCTCTGGCCTAGAGGACAAGGTTTCAAAGCAGTTGGAATCAAAAGGTATTAAATTCGAGTATGAAGAGTGGAAAGTGCCTTATGTAATTCCGGCGAGCAATCACACTTACACTCCAGACTTCTTACTTCCAAACGGTATATTCGTTGAGACAAAGGGTCTGTGGGAAAGCGATGATAGAAA    >  1:202317/1‑301`  
                                                                                                                                                                                                                                                                                                           `GTCCAATGTATCCTTAGCTCGCACCTATGGTGTCTCCCAACAGACTATTTGTGATATACGCAAAGGGAGGCGACATGGCAGGTTACGGCGCTAAAGGAATCCGAAAGGTTGGAGCGTTTCGCTCTGGCCTAGAGGACAAGGTTTCAAAGCAGTTGGAATCAAAAGGTATTAAATTCGAGTATGAAGAGTGGAAAGTGCCTTATGTAATTCCGGCGAGCAATCACACTTACACTCCAGACTTCTTACTTCCAAACGGTATATTCGTTGAGACAAAGGGTCTGTGGGAAAGCGATGATAGAAA    >  1:439223/1‑301`  
                                                                                                                                                                                                                                                                                                           `GTCCAATGTATCCTTAGCTCGCACCTATGGTGTCTCCCAACAGACTATTTGTGATATACGCAAAGGGAGGCGACATGGCAGGTTACGGCGCTAAAGGAATCCGAAAGGTTGGAGCGTTTCGCTCTGGCCTAGAGGACAAGGTTTCAAAGCAGTTGGAATCAAAAGGTATTAAATTCGAGTATGAAGAGTGGAAAGTGCCTTATGTAATTCCGGCGAGCAATCACACTTACACTCCAGACTTCTTACTTCCAAACGGTATATTCGTTGAGACAAAGGGTCGGTGGGAAAGCGATGCTAGAta    >  1:163620/1‑299`  
                                                                                                                                                                                                                                                                                                           `GTCCAATGTATCCTTAGCTCGCACCTATGGTGTCTCCCAACAGACTATTTGTGATATACGCAAAGGGAGGCGACATGGCAGGTTACGGCGCTAAAGGAATCCGAAAGGTTGGAGCGTTTCGCTCTGGCCTAGAGGACAAGGTTTCAAAGCAGTTGGAATCAAAAGGTATTAAATTCGAGTATGAAGAGTGGAAAGTGCCTTATGTAATTCCGGCGAGCAATCACACTTACACTCCAGACTTCTTACTTCCAAACGGTATATTAGTTGAGACAAAGGGTCctgtctcttatacacatctccc    >  1:265319/1‑279`  
                                                                                                                                                                                                                                                                                                           `GTCCAATGTATCCTTAGCTCGCACCTATGGTGTCTCCCAACAGACTATTTGTGATATACGCAAAGGGAGGCGACATGGCAGGTTACGGCGCTAAAGGAATCCGAAAGGTTGGAGCGTTTCGCTATGGCCTAGAGGACAAGGTTTCAAAGCAGTTGGAATCAAAAGGTATTAAATTCGAGTATGAAGAGTGGAAAGTGCCTTCTGTAATTCCGGCGAGCAATCACACTTACACTCCAGACTTCTTACTTCCAAACGGTATATTCGTTGCGACAAAGTGTCTGTGGGAAAGCGCTGATAGAAA    >  1:262720/1‑301`  
                                                                                                                                                                                                                                                                                                           `GTCCAATGTATCCTTAGCTCGCACCTATGGTGTCTCCCAACAGACTATTTGTGATATACGCAAAGGGAGGCGACATGGCAGGTTACGGCGCTAAAGGAATCCGAAAGGTTGGAGCGTTTCGATCTGGCCTAGAGGACAAGGTTTCAAAGCAGTTGGAATCAAAAGGTATTAAATTCGAGTATGAAGAGTGGAAAGTGCCTTATGTAATTCCGGCGAGCAATCACACTTACACTCCAGACTTCTTACTTCCAAACGGTATATTCGTTGAGAAAAAGGGTCTGTGGGAAAGCGATGATAGAAA    >  1:148581/1‑301`  
                                                                                                                                                                                                                                                                                                           `GTCCAATGTATCCTTAGCTCGCACCTATGGTGTCTCCCAACAGACTATTTGTGATATACGCAAAGGGAGGCGACATGGCAGGTTACGGCGCTAAAGGAATCCGAAAGGTTGGAGCATTTCGCTCTGGCCTAGAGGACAAGGTTTCAAAGCAGTTGGAATCAAAAGGTATTAAATTCGAGTATGAAGAGTGGAAAGTGCCTTATGTAATTCCGGCGAGCAATCACACTTACACTCCAGACTTCTTACTTCCAAACGGTATATTCGTTGAGACAAAGGTTATGTGGGAAAGCGATGATAGAAA    >  1:50620/1‑301`  
                                                                                                                                                                                                                                                                                                           `GTCCAATGTATCCTTAGCTCGCACCTATGGTGTCTCCCAACAGACTATTTGTGATATACGCAAAGGGAGGCGACATGGCAGGTTACGGCGCTAAAGGAATCCGAAAGGTTGGAGAGTTTCGCTCTGGCCTAGAGGACAAGGTTTCAAAGCAGTTGGAATCAAAAGGTATTAAATTCGAGTATGAAGAGTGGAAAGTGCCTTATGTAATTCCGGCGAGCAATCACACTTACACTCCAGACTTCTTACTTCCAAACGGTATATTCGTTGAGACAAAGGGTCTGTGGGAAAGCGATGATAGAAA    >  1:444951/1‑301`  
                                                                                                                                                                                                                                                                                                           `GTCCAATGTATCCTTAGCTCGCACCTATGGTGTCTCCCAACAGACTATTTGTGATATACGCAAAGGGAGGCGACATGGCAGGTTACGGCGCTAAAGGAATCAGAAAGGTTGGAGCGTTTCGCTCTGGCCTAGAGGACAAGGTTTCAAAGCAGTTGGAATCAAAAGGTATTAAATTCGAGTATGAAGAGTGGAAAGTGCCTTATGTAATTCCGGCGAGCAATCACACTTACACTCCAGACTTCTTACTTCCAAACGGTATATTCGTTGAGACAAAGGGTCTGTGGGAAAGCGAatgtctctt    >  1:357512/1‑292`  
                                                                                                                                                                                                                                                                                                           `GTCCAATGTATCCTGAGCTCGCACCTATGGTGTCTCCAAACAGACTATTTGTGATATACGCAAAGGGAGGCGACATGGCAGGTTACGGCGCTAAATGAATCCGAAAGGTTGGAGCGTTTCGCTCTGGCCTAGAGGACAAGGTTTCAAAGCAGTTGGAATCAAAAGGTATTAAATTCGAGTATGAAGAGTGGAAAGTGCCTTATGTAATTCCGGCGAGCAATCACACTTACACTCCAGACTTCTTACTTCCAAACGGTATATTCGTTGAGACAAAGGGTCTGTGGGCAAGCGATGATAGAAA    >  1:252260/1‑301`  
                                                                                                                                                                                                                                                                                                            `TCCAATGTATGATTAGATCGCACCTATGGTGTCTCCCAACAGACTATTTGTGATATACGCAAAGGGAGGCGACATGGCAGGTTACGGCGCTAAAGGAATCCGAAAGGTTGGAGCGTTTCGCTCTGGCCTAGAGGACAAGGTTTCAAAGCAGTTGGAATCAAAAGGTATTAAATTCGAGTATGAAGAGTGGAAAGTGCCTTATGTAATTCCGGCGAGCAATCACACTTACACTCCAGACTTCTTACTTCCAAACGGTATATTCGTTGAGACAAAGGGTCTGTGGGAAAGCGATGATAGAAAG   <  1:330236/301‑1`  
                                                                                                                                                                                                                                                                                                            `TCCAATGTATCCTTAGCTCGCACCTATGGTGTCTCCCAACAGACTATTTGTGATATACGCAAAGGGAGGCGACATGGCAGGTTACGGCGCTAAAGGAATCCGAAAGGTTGGAGCGTTTCGCTCTGGCCTAGAGGACAAGGTTTCAAAGCAGTTGGAATCAAAAGGTATTAAATTCGAGTATGAAGAGTGGAAAGTGCCTTATGTAATTCCGGCGAGCAATCACACTTACACTCCAGACTTCTTACTTCCAAACGGTATATTCGTTGAGACAAAGGGTCTGTGGGAAAGCGATGATAGAAAG   <  1:239447/301‑1`  
                                                                                                                                                                                                                                                                                                            `TCCAATGTATCCTTAGCTCGCACCTATGGTGTCTCCCAACAGACTATTTGTGATATACGCAAAGGGAGGCGACATGGCAGGTTACGGCGCTAAAGGAATCCGAAAGGTTGGAGCGTTTCGATCTGGCCTAGAGGACAAGGTTTCAAAGCAGTTGGAATCAAAAGGTATTAAATTCGAGTATGAAGAGTGGAAAGTGCCTTATGTAATTCCGGCGAGCAATCACACTTACACTCCAGACTTCTTACTTCCAAACGGTATATTCGTTGAGACAAAGGGTCTGTGGGAAAGCGATGATAGAAAG   <  1:152499/301‑1`  
                                                                                                                                                                                                                                                                                                             `CCAATGTCTCGTTAGCCGGAACCATTGGGTTTTCCCAGAAGAGTATGTGTGATATACTCAAAGGGAGGCGACATGGCAGGTTACGGCGCTAAAGGAATCCGAAAGGTTGGAGCGTGTCGCTATGGCCTAGAGGACAAGGTTTCAAAGCAGTTTGAATCAAAAGGTCTTAAATTCGAGTATGAATAGTGGAAAGTGCCTTATGTAATTCCGGCGAGCAATCACACTTACACTCCAGACTTCTTACTTCCAAACGGTATATTCGTTGAGACAAAGGGTCTGTGGGAAAGCGATGATAGAAAGA  <  1:364092/301‑1`  
                                                                                                                                                                                                                                                                                                             `CCAATGTATCCTTCGCTAGCACCTATGGTGTCTCCAAACAGACTGTTTGTGATATACGAAAAGGGAGGCGACATGGCAGGTTACGGCGCTAAATGAATCCGAAAGGTTGGAGCGTTTCGCTCTGGCCTAGAGGACAAGGTTTCAAAGCAGTGGGAATCAAAAGGTATTAAATTCGAGTATGAAGAGTGGAAAGTGCCTTATGTAATTCCGGCGAGCAATCACACTTACACTCCAGACTTCTTACTTCCAAACGGTATATTCGTTGAGACAAAGGGTCTGTGGGAAAGCGATGATAGAAAGA  <  1:12952/301‑1`  
                                                                                                                                                                                                                                                                                                             `CCAATGTATCCTTAGTTCGGATCTATGTTGTCTCCCAACAGACTATTTTTGTTATACGCAAAGGGATGCGACATGGCAGGTTACTGCGCTAAATGAATCCGAAAGGTTGGAGCGTTTCGCTCTGGCCTAGAGGACAATGTTTCAAAGCAGTTGGAATCAAAAGGTATTAAATTCGAGTATGAAGAGTGGAAAGTGCCTTATGTAATTCCGGCGAGCAATCACACTTACACTCCAGACTTCTTACTTCCAAACGGTATATTCGTTGAGACAAAGGGTCTGTGGGAAAGCGATGATAGAAAGA  <  1:448870/301‑1`  
                                                                                                                                                                                                                                                                                                             `CCAATGTATCCTTAGCTCGCACCTATGGTGTCTCCCAACGGACTATTTGTGATATACGCAAATGGAGGCGACATGGCAGGTTACGGCGCTAAAGGAATCCGAAAGGTTGGAGCGTTTCGCTCTGGCCTAGAGGACAAGGTTTCAAAGCAGTTGGAATCAAAAGGTATTAAATTCGAGTATGAAGAGTGGAAAGTGCCTTATGTAATTCCGGCTAGCAATCACACTTACACTCCAGACTTCTTACTTCCACACGGTATATTAGTTGCGACACAGGGTCTGTTGGAAAGCGATGATAGAAAGA  >  1:85648/1‑301`  
                                                                                                                                                                                                                                                                                                             `CCAATGTATCCTTAGCTCGCACCTATGGTGTCTCCCAACAGACTATTTGTGATATACGCAAAGGGAGGCGACATGGCAGGTTACGGCGCTAAAGGAATCCGAAAGGTTGGAGCGTTTCGCTCTGGCCTAGAGGACAAGGTTTCAAAGCAGTTGGAATCAAAAGGTATTAAATTCGAGTATGAAGAGTGGAAAGTGCCTTATGTAATTCCGGCGAGCAATCACACTTACACTCCAGACTTCTTACTTCCAAACGGTATATTCGTTGAGACAAAGGGTCTGTGGGAAAGCGATGATAGAAAGA  <  1:270479/301‑1`  
                                                                                                                                                                                                                                                                                                             `CCAATGTATCCTTAGCTCGCACCTATGGTGTCTCCCAACAGACTATTTGTGATATACGCAAAGGGAGGCGACATGGCAGGTTACGGCGCTAAAGGAATCCGAAAGGTTGGAGCGTTTCGCTCTGGCCTAGAGGACAAGGTTTCAAAGCAGTTGGAATCAAAAGGTATTAAATTCGAGTATGAAGAGTGGAAAGTGCCTTATGTAATTCCGGCGAGCAATCACACTTACACTCCAGACTTCTTACTTCCAAACGGTATATTCGTTGAGACAAAGGGTCTGTGGGAAAGCGATGATAGAAAGA  >  1:303707/1‑301`  
                                                                                                                                                                                                                                                                                                             `CCAATGTATCCTTAGCTCGCACCTATGGTGGCTCACAACAGACTATTTGGGATATAAGCAAAGGGAGGCGAAATGGCAGGTTACGGCGATAAAGGAATCCGAAAGGTTGGAGCGTTTCGCTCTGGCCTAGAGGACAAGGTTTCAAAGCAGTTGGAATCAAAAGGTATTAAATTCGAGTATGAAGAGTGGAAAGTGCCTTATGTAATTCCGGCGAGCAATCACACTTACACTCCAGACTTCTTACTTCCAAACGGTATATTCGTTGAGACAAAGGGTCTGTGGGAAAGCGATGATAGAAAGA  <  1:243347/301‑1`  
                                                                                                                                                                                                                                                                                                             `CCAATGTATCCATAGCTCGCATCTATTGTGTCTCCCAACAGACTATTTGTGATATACGCAAAGGGAGGCGACATGGCAGGTTACGGCTCTAAAGGAATCCGAAAGGTTGGAGCGTTTCGCTCTGGCCTAGAGGACAAGGTTTCAAAGCAGTTGGAATCAAAAGGTATTAAATTCGAGTATGAAGAGTGGAAAGTGCCTTATGTAATTCCGGCGAGCAATCACACTTACACTCCAGACTTCTTACTTCCAAACGGTATATTCGTTGAGACAAAGGGTCTGTGGGAAAGCGATGATAGAAAGA  <  1:344526/301‑1`  
                                                                                                                                                                                                                                                                                                             `CCAATGTAGCCTTAGCTCGCAACTATGGTGTCTCCCAACAGGCTATTTGTGAGATACGCAAAGGGAGGCGACATGGCAGGTTACGGCGCTAAAGGAATCCGAAAGGTTGGAACGTTTCGCTATGGCCTAGAGGAAAAGGTTTAAAAGCAGTTGGAATCAAAAGGTATTAAATGTGAGGATGAAGAGTGGAAAGTGCATTATGTAAATCCGGCGAGCAATCACAATTAAACTCCAGACTTCTTACGTAAAAACGGTATATGCGTTGAGACAAAGGGTCTGTGGGAAAGCGATGATAGAAAGA  <  1:355941/301‑1`  
                                                                                                                                                                                                                                                                                                             `CCAATCTATCCTTAGCTCGCAACTATGGTGTCGTCAAAAAGACGATTTGTGATATACGAAAAGGGAGGCGAAAGGGCAGGTGACGGCGCTAAAGGAATCAGAAAGGTTTGAGCGTTTCGCTCTTGCCTAGAGGACAAGGTTGAAAAGCAGTTGGAATCAAAAGGTATTAAATTCGAGTATGAAGAGTGGAAAGTGCCTTATGTAATTCCGGCGAGCAATCACACTTACACTCCAGACTTCTTACTTCCAAACGGTATATTCGTTGAGACAAAGGGTCTGTGGGAAAGCGATGATAGAAAGA  <  1:46850/301‑1`  

                                                                                                                                                                                                                                                                                                                                                                                                                                                                                                                                                                                                                            
`ACCTTTGGTGGCGGTGAAGACGATTGGGCTGACGAAGTTGAAGAGAACGGCTATGTTGCCTCTGGTTCTGCCAAAGCGAGCAAACCACGCGACGAAGAAAGCTGGGACGAAGACGACGAAGAGTCCGAGGAAGCAGACGAAGACGGAGACTTCTAAGTGGAACTGCGGGAGAAAATCCTTGAGCGAATCAAGGTGACTTCCTCTGGGTGTTGGGAGTGGCAGGGCGCTACGAACAATAAAGGGTACGGGCAGGTGTGGTGCAGCAATACCGGAAAGGTTGTCTACTGTCATCGCGTAATGTC‑‑‑‑‑‑‑‑‑‑‑‑‑‑‑‑‑‑‑‑‑‑‑‑‑‑‑‑‑‑‑‑‑‑‑‑‑‑‑‑‑‑‑‑‑‑‑‑‑‑‑‑‑‑‑‑‑‑‑‑‑‑‑‑‑‑‑‑‑‑‑‑‑‑‑‑‑‑‑‑‑‑‑‑‑‑‑‑‑‑‑‑‑‑‑‑‑‑‑‑‑‑‑‑‑‑‑‑‑‑‑‑‑‑‑‑‑‑‑‑‑‑‑‑‑‑‑‑‑‑‑‑‑‑‑‑‑‑‑‑‑‑‑‑‑‑‑‑‑‑‑‑‑‑‑‑‑‑‑‑‑‑‑‑‑‑‑‑‑‑‑‑‑‑‑‑‑‑‑‑‑‑‑‑‑‑‑‑‑‑‑‑‑‑‑‑‑‑‑‑‑‑‑‑‑‑‑‑‑‑‑‑‑‑‑‑‑‑‑‑‑‑‑‑‑‑‑‑‑‑‑‑‑‑‑‑‑‑‑‑‑‑‑‑‑‑‑‑‑‑‑‑‑‑‑‑‑‑‑‑‑‑‑‑‑‑‑‑‑‑‑‑‑‑‑‑‑‑‑‑‑‑‑‑‑‑‑‑‑‑‑‑‑‑‑‑‑‑‑‑  >  NC_001604.1/9701‑10002`  
`‑‑‑‑‑‑‑‑‑‑‑‑‑‑‑‑‑‑‑‑‑‑‑‑‑‑‑‑‑‑‑‑‑‑‑‑‑‑‑‑‑‑‑‑‑‑‑‑‑‑‑‑‑‑‑‑‑‑‑‑‑‑‑‑‑‑‑‑‑‑‑‑‑‑‑‑‑‑‑‑‑‑‑‑‑‑‑‑‑‑‑‑‑‑‑‑‑‑‑‑‑‑‑‑‑‑‑‑‑‑‑‑‑‑‑‑‑‑‑‑‑‑‑‑‑‑‑‑‑‑‑‑‑‑‑‑‑‑‑‑‑‑‑‑‑‑‑‑‑‑‑‑‑‑‑‑‑‑‑‑‑‑‑‑‑‑‑‑‑‑‑‑‑‑‑‑‑‑‑‑‑‑‑‑‑‑‑‑‑‑‑‑‑‑‑‑‑‑‑‑‑‑‑‑‑‑‑‑‑‑‑‑‑‑‑‑‑‑‑‑‑‑‑‑‑‑‑‑‑‑‑‑‑‑‑‑‑‑‑‑‑‑‑‑‑‑‑‑‑‑‑‑‑‑‑‑‑‑‑‑‑‑‑‑‑‑‑‑‑‑‑‑‑‑‑‑‑‑‑‑‑‑‑‑‑‑‑‑‑‑‑‑‑‑‑‑‑‑‑gtcCAATGTATCCTTAGCTCGCACCTATGGTGTCTCCCAACAGACTATTTGTGATATACGCAAAGGGAGGCGACATGGCAGGTTACGGCGCTAAAGGAATCCGAAAGGTTGGAGCGTTTCGCTCTGGCCTAGAGGACAAGGTTTCAAAGCAGTTGGAATCAAAAGGTATTAAATTCGAGTATGAAGAGTGGAAAGTGCCTTATGTAATTCCGGCGAGCAATCACACTTACACTCCAGACTTCTTACTTCCAAACGGTATATTCGTTGAGACAAAGGGTCTGTGGGAAAGCGATGATAGAAAGA  >  NC_001604.1/10186‑10485`

  

Alignment Legend

Aligned base mismatch/match (shaded by quality score): `ATCG/ATCG` `< 3 ≤` `ATCG/ATCG` `< 9 ≤` `ATCG/ATCG` `< 21 ≤` `ATCG/ATCG` `< 34 ≤` `ATCG/ATCG` `< 38 ≤` `ATCG/ATCG`

Unaligned base: `atcg`    Masked matching base: `atcg`    Alignment gap: `‑`    Deleted base: `‑`

Reads not counted as support for junction

read\_name Not counted due to insufficient overlap past the breakpoint.

read\_name Not counted due to not crossing MOB target site duplication.
